# Conflicts of interest in biomedical publications: considerations for authors, peer reviewers, and editors

**DOI:** 10.3325/cmj.2013.54.600

**Published:** 2013-12

**Authors:** Armen Yuri Gasparyan, Lilit Ayvazyan, Nurbek A. Akazhanov, George D. Kitas

**Affiliations:** 1 Departments of Rheumatology and Research and Development, Dudley Group NHS Foundation Trust (A Teaching Trust of The University of Birmingham), Russells Hall Hospital, Dudley, United Kingdom *a.gasparyan@gmail.com*; 2 Department of Medical Chemistry, Yerevan State Medical University, Yerevan, Armenia; 3 Department of Internship and Residency for General Practitioners N3, Kazakh National Medical University, Almaty, Kazakhstan; 4 Arthritis Research UK Epidemiology Unit, University of Manchester, Manchester, United Kingdom

## Abstract

This article overviews evidence on common instances of conflict of interest (COI) in research publications from general and specialized fields of biomedicine. Financial COIs are viewed as the most powerful source of bias, which may even distort citation outcomes of sponsored publications. The urge to boost journal citation indicators by stakeholders of science communication is viewed as a new secondary interest, which may compromize the interaction between authors, peer reviewers, and editors. Comprehensive policies on disclosure of financial and non-financial COIs in scholarly journals are presented as proxies of their indexing in evidence-based databases, and examples of successful medical journals are discussed in detail. Reports on clinical trials, systematic reviews, meta-analyses, and clinical practice guidelines may be unduly influenced by author-pharmaceutical industry relations, but these publications do not always contain explicit disclosures to allow the readers to judge the reliability of the published conclusions and practice-changing recommendations. The article emphasizes the importance of adhering to the guidance on COI from learned associations such as the International Committee of Medical Journal Editors (ICMJE). It also considers joint efforts of authors, peer reviewers, and editors as a foundation for appropriately defining and disclosing potential COIs.

Conflict of interest (COI), or competition of interests, often distorts presentation and interpretation of research data in biomedical publications, and its non-disclosure is perceived as a misconduct with serious consequences for trustworthiness of science communication (1).

In 1991, the term COI was introduced in the Medical Subject Headings (MeSH) of the National Library of Medicine of the US, and 10 200 entries have been tagged with this term in PubMed as of December 5, 2013. An increasing body of related research and scientific publications, with just one PubMed-indexed item on COI in 1967 and 670 items in 2010, speaks volumes about the growing importance of the subject for the multidisciplinary medical community. However, elaborations on COI across subspecialty areas are uneven and often scarce. For example, PubMed lists only 296 (2.9%) relevant sources in internal medicine, 113 (1.1%) in cardiology, and only 17 (0.2%) in rheumatology.

Although no unified definition of COI exists for the medical community, it is widely described as a set of circumstances in which a primary professional interest is excessively influenced by an individual’s secondary interest(s), which come into conflict with ethical duties toward patients, health professionals, and society-at-large (2,3).

In medicine, primary professional interests relate to the quality of health care, proper management of diseases, well-being of patients, professional service to a discipline and a community, unbiased execution and reporting of scientific research, as well as honest and transparent editorial work. Secondary interests are numerous, complex in origin, and not always easily discernible, even for seasoned experts. These may arise from an individual’s desire to benefit financially from professional actions, to strengthen positions of certain scientific statements, to advance his/her career development, or to favor family, friends, and colleagues from the same city, country, or institution, among others. The resultant conflict may be commercial (financial), intellectual, academic (academic competition), ideological, personal, or regional.

Financial relationships of research institutions and their investigators is the most conspicuous source of conflict. An individual’s sources of financial conflicts include, but are not limited to grants, stock ownership, honoraria, speaker fees, royalties, and salary. In quantitative terms, research funding authorities in the US consider a threshold of US $10 000 a year or 5% ownership in any commercial entity as “a significant source of conflict” that may subjectively affect research (4). Alas, one of the landmark studies on financial COIs in randomized clinical trials reported in the *BMJ* from 1997 to 2001 (159 papers from 12 specialties) revealed that the authors’ conclusions were significantly more positive toward pharmacological or non-pharmacological interventions in trials funded by for-profit organizations than in those without any such conflict (*P* = 0.014) (5). The same study claimed that personal and academic conflicts did not affect authors’ conclusions. Analysis of citation outcomes of 303 cardiovascular trials reported in the *Journal of the American Medical Association*, *The Lancet,* and *The New England Journal of Medicine* from 2000 to 2005 indicated a strikingly high citation rate of trials funded by for-profit organizations, excessively investing in open access, secondary publications, and wide dissemination of the reports (6).

The current peer review system is likely to be affected by secondary interests of expert reviewers, who support or criticize manuscripts, or push citations to their works on subjective, non-scientific grounds (7). Dual or multiple competing affiliations, academic interests, and financial ties of peer reviewers with the pharmaceutical industry are serious threats to the objectivity of experts’ judgments and decisions throughout the peer review process, especially in small professional communities (1).

A relatively new secondary interest that can be attributed to the shortcomings of “the big science” era is the urge to improve prestige, productivity, and citation profiles of a journal. Editors, who take multiple decision-making posts in competing journals, push citations to “friendly” articles and create “citation stacking schemes,” are particularly exposing themselves to conflicts that undermine the validity of the editorial work (8). The urge to improve journal ranks also creates a series of hurdles for editors and publishers, who may be tempted to publish potentially citable papers circumventing rigorous peer review. They often prioritize reports on trials, large cohort studies, and clinical recommendations that are heavily influenced by the authors’ financial and personal relations with pharmaceutical industry and other funding organizations. As a result, the emerging interest in boosting citations threatens to compromise the interaction between authors, reviewers, and editors.

All types of journals, including publishing outlets of professional societies, established and newly-launched national and international periodicals, may suffer from the lack of awareness of the issue of COI among science editors and its inappropriate disclosure by all stakeholders of the science communication. For newly-launched, small, and non-mainstream science journals, the editors’ commitment to accurate and transparent disclosure of financial and non-financial COIs can help improve quality of the editorial work and pave the way for journal indexing in major evidence-based bibliographic databases (9-11). In contrast, partial or inappropriate disclosure of COIs can be detrimental for scientific prestige of well-established and influential journals, where numerous therapeutic agents, promoted by large pharmaceutical agencies, are discussed and get their approval for the long-term management of disabling diseases (12). Notably, a pioneering survey among a group of editors of general and internal medicine journals with published impact factors, conducted by the *BMJ* editorial staff in 2004, claimed that only 9 out of 30 surveyed journals (30%) had established an explicit policy to deal with the editors’ financial COIs, while 12 editors (37%) did not intend to declare financial conflicts in the future (13). The status of COI was better in top-tier journals such as *The New England Journal of Medicine, JAMA,* and *The Lancet* than in lower-impact periodicals. Surprisingly, disclosure of the editorial board members’ and editorial advisers’ financial and non-financial conflicts were viewed by most respondents as not important (13). Furthermore, a more recent survey of editors of 46 cardiovascular and allied journals affiliated to the European Society of Cardiology, revealed a lack of a systematic approach to the issue of authors’, reviewers’, and editors’ COI (14). Specific policies on authors’ COI were in place only in 20 (44%), on reviewers’ COI in 11 (25%), and on editors’ COI in 8 journals (18%); and only 15 (36%) respondent editors were familiar with the widely circulated COI disclosure form of the International Committee of Medical Journal Editors (ICMJE).

Rheumatology is another rapidly evolving clinical discipline, where over the past 2 decades a variety of new biological agents with targeted immunomodulatory properties have improved treatment outcomes of rheumatic disorders. There may obviously be a strong financial interest in outcomes of research on numerous therapeutic agents and their interpretation in original, review, and clinical practice guideline (CPG) articles, which are increasingly published in rheumatology journals. However, like in the field of cardiovascular medicine, policies on COI in rheumatology journals are still imperfect. We reviewed webpages, instructions for authors, and publishers’ policies on COI of 43 Scopus-indexed rheumatology journals, currently listed in the SCImago database, and found that only 7 (16.3%) have adopted comprehensive policies on COI disclosure for authors, reviewers, and editors. All these journals are distinguished by high scientific prestige, high values of the *h* index and the journal impact factor. Of the 43 journals, 30 (69.8%) have still not declared their policies for transparent reporting of COI among the reviewers and editors (Table 1).

**Table 1:** Policies on reporting authors’, reviewers’, and editors’ conflict of interests in rheumatology journals*

N	Abbreviated Journal Titles	SJR quartile	H index	2-Y JIF	Authors	Reviewers	Editors
1	*Ann Rheum Dis*	Q1	132	9.111	+	+	+
2	*Arthritis Rheum*	Q1	211	7.477	+	+	+
3	*Arthritis Res Ther*	Q1	84	4.302	+	+	+
4	*Arthritis Care Res*	Q1	82	3.731	+	+	+
5	*Nat Rev Rheumatol*	Q1	52	9.745	+	+	+
6	*Rheumatology*	Q1	106	4.212	+	+	+
7	*Semin Arthritis Rheum*	Q1	73	3.806	+	+	+
8	*Clin Exp Rheumatol*	Q1	62	2.655	+	NA	NA
9	*Rheum Dis Clin North Am*	Q1	61	2.096	+	+	NA
10	*Curr Rheumatol Rep*	Q1	37	-	+	NA	NA
11	*BMC Musculoskelet Dis*	Q1	41	1.875	+	+	+
12	*Joint Bone Spine*	Q2	43	2.748	+	NA	NA
13	*Musculoskelet Care*	Q2	12	-	NA	NA	NA
14	*Rheumatol int*	Q2	43	2.214	+	NA	NA
15	*Bull NYU Hosp Jt Dis*	Q2	26	-	+	NA	NA
16	*Biologics*	Q2	12	-	+	+	NA
17	*J Clin Rheumatol*	Q2	29	1.183	NA	NA	NA
18	*Pediatr Rheumatol*	Q2	10	1.47	+	+	+
19	*Int J Rheum Dis*	Q2	12	1.65	+	NA	NA
20	*Reumatismo*	Q2	13	-	+	NA	NA
21	*Open Rheumatol J*	Q2	3	-	+	NA	NA
22	*Int J Clin Rheumatol*	Q2	6	-	+	+	+
23	*Acta Reumatol Port*	Q3	10	0.695	+	NA	NA
24	*Rev Bras Ruematol*	Q3	10	-	+	NA	NA
25	*Int J Adv Rheumatol*	Q3	3	-	NA	NA	NA
26	*Curr Rheumatol Rev*	Q3	7	-	+	NA	NA
27	*Autoimmunity Highlights*	Q3	3	-	+	NA	NA
28	*Reumatol Clin*	Q3	7	-	+	NA	NA
29	*J Musculoskelet Pain*	Q3	25	0.328	+	NA	NA
30	*Z Rheumatol*	Q3	31	0.450	+	NA	NA
31	*Rheumatol Rep*	Q3	1	-	+	NA	NA
32	*Turk J Rheumatol*	Q3	3	0.172	+	NA	NA
33	*Rev Rhum Monograph*	Q3	3	-	+	NA	NA
34	*Open Access Rheumatol*	Q4	3	-	+	+	NA
35	*Ceska Revmatol*	Q4	6	-	NA	NA	NA
36	*Reumatologia*	Q4	7	-	+	NA	NA
37	*Ther Adv Muskuloskelet Dis*	Q4	2	-	+	NA	NA
38	*Open Arthritis J*	Q4	0	-	+	NA	NA
39	*Indian J Rheumatol*	Q4	5	-	+	NA	NA
40	*Akt Rheumatol*	Q4	9	0.097	+	NA	NA
41	*Rev Rhum (Edition Francaise)*	Q4	28	-	+	NA	NA
42	*Semin Fund Esp Reumatol*	Q4	2	-	+	NA	NA
43	*Reumatol Clin Supl*	Q4	3	-	NA	NA	NA

*Data are obtained from the SCImago Journal and Country Rank database (SCImago Journal Rank [SJR] quartiles and journal *H* index values for 2013) and the Journal Citation Reports 2013 (2-Year Journal Impact Factors [2-Y JIF]). NA – not available.

With the current pace of digitization and systematization of online searches through bibliographic databases, it is likely that inappropriate disclosure of COIs in primary research studies, published in any peer-reviewed and indexed journal, will ultimately impair the trustworthiness of the evidence synthesis and expert statements in reviews and CPGs. Indeed, a comprehensive analysis of search strategies and COI reporting in 281 narrative and systematic reviews that focused on the widely used anti-rheumatic biologics, infliximab and etanercept, pointed to the poor adherence to research reporting guidelines and the lack of primary source validation in most articles, even in publications in high-impact rheumatology journals (15). Conflict disclosure was at unacceptably low levels in both types of reviews, even though the systematic ones displayed detailed COI notes more often (25% vs 42%; *P* < 0.005) (15). Similar inaccurate and biased reporting also takes place in many other areas of clinical medicine, especially when primary data from randomized trials on drug interventions are pooled and processed in systematic reviews and meta-analyses (16,17). As a prime example, of the 151 items published in the Cochrane Database of Systematic Reviews in 2010, only 46 (30%) provided statements on funding sources of the overviewed trials and 16 (11%) on trial author-industry financial ties and employment (17).

The Cochrane reviews often reinforce CPGs and thus become guiding tools for a large number of practicing physicians. Authors of these guidelines may have financial and other relations with the manufacturers of the drugs they recommend, and any such instance, especially when concealed or inexplicitly reported, may jeopardize the validity of the guidelines as intervention tools for health care worldwide (18). Peer reviewers and editors of the highest impacting journals, where the CPGs are usually published, should be aware of the prevalence and consequences of the conflicting relations of sponsors and authors of the CPGs for the global medical community. In 2002, the earliest survey of 100 authors of 37 guidelines on common adult diseases, endorsed by major North American and European societies, indicated that 59% of the authors had conflicting relations with pharmaceutical companies whose drugs were discussed in the documents they authored (19). Surprisingly, the same percentage of respondents claimed that the disclosure of financial and non-financial relations with these companies was not obligatory during the guideline development (19). A recent study from the National Guideline Clearinghouse, a database of evidence-based CPGs of the US Department of Health and Human Services, explored specific author-industry relations in 13 major guidelines on hypoglycemic drugs for type 2 diabetes and uncovered that 56% of manufacturers of drugs discussed in each guideline had direct financial ties with the authors, while three of the guidelines did not contain a disclosure of such relations at all (20).

Authors of most practice guidelines are usually eminent specialists in their field, who are sponsored by one or more pharmaceutical agencies. High-ranking journals are much desirable publication venues for the authors and sponsors who aim to attract large numbers of readers and potential citers to the CPGs. Unfortunately, disclosure of COIs is still not a top priority throughout the development and distribution of the guidelines. Our experience with processing relevant documents, retrieved from PubMed/MEDLINE (21-47), suggests that the field of rheumatology is no exclusion (Table 2). The absolute majority of CPGs in rheumatology are about new anti-rheumatic drugs. The guidelines are often written and supported by leading members of the European League Against Rheumatism (EULAR), with a solid publication record in the *Annals of the Rheumatic Diseases*, the official organ of the Association with impressive bibliometric indicators. Analysis of 27 guidelines, endorsed by EULAR and published from 2000 to 2013, indicates that COI statements are available in only 9 (33.3%) documents, while 18 (66.6%) do not contain detailed disclosures of COIs and author-industry specific relations. However, COI notes in the six most recent EULAR recommendations (2010-2013) contain exhaustive disclosure of pharmaceutical industry-related conflicts. This trend may indicate improved understanding of the importance of COI among authors, reviewers, and handling editors of the CPGs.

**Table 2:** Conflict of interest notes and explicit disclosures of author relations with manufacturers of drugs and medical technologies recommended in rheumatology practice guidelines*

Subject of practice guidelines	Conflict of interest disclosures	Disclosures of specific author-industry relations	References
Pharmacological and non-pharmacological therapies in knee osteoarthritis	NA	NA	Pendleton A et al, 2000 (21)
Pharmacological and non-pharmacological therapies in hip osteoarthritis	NA	NA	Zhang W et al, 2005 (22)
Biological and non-biological drug therapies in ankylosing spondylitis	NA	NA	Zochling J et al, 2006 (23)
Systemic glucocorticoid therapy in rheumatic diseases	NA	NA	Hoes JN et al, 2007 (24)
Drug therapies in hand osteoarthritis	NA	NA	Zhang W et al, 2007 (25)
Non-biological drug therapies in early rheumatoid arthritis	NA	NA	Combe B et al, 2007 (26)
Biological and non-biological therapies in Behçet disease	NA	NA	Hatemi G et al, 2008 (27)
Pharmacological and non-pharmacological therapies in fibromyalgia	+	+	Carville SF et al, 2008 (28)
Drug therapies in lupus	NA	NA	Bertsias G et al, 2008 (29)
Diagnosis of hand osteoarthritis	NA	NA	Zhang W et al, 2009 (30)
Biological and non-biological therapies in ankylosing spondylitis	NA	NA	Kiltz U et al, 2009 (31)
Biological and non-biological therapies in rheumatoid arthritis	+	+	Smolen JS et al, 2010 (32)
Cardiovascular and anti-inflammatory drug therapies in rheumatic diseases	NA	NA	Peters MJ et al, 2010 (33)
Biological and non-biological therapies in neuropsychiatric lupus	NA	NA	Bertsias GK et al, 2010 (34)
Vaccinations in pediatric patients with rheumatic diseases	NA	NA	Heijstek MW et al, 2011 (35)
Vaccinations in adults with rheumatic diseases	NA	NA	van Assen S et al, 2011 (36)
Biological and non-biological drug therapies in axial spondyloarthritis	NA	NA	van der Heijde D et al, 2011 (37)
Drug therapies in calcium pyrophosphate deposition	NA	NA	Zhang W et al, 2011 (38)
Biological and non-biological therapies in ankylosing spondylitis	NA	NA	Braun J et al, 2011 (39)
Drug therapies in gout and hyperuricemia	+	+	Hamburger M et al, 2011 (40)
Biological and non-biological drug therapies in lupus nephritis	NA	NA	Bertsias GK et al, 2012 (41)
Biological and non-biological drug therapies in psoriatic arthritis	+	+	Gossec L et al, 2012 (42)
Non-pharmacological management of hip and knee osteoarthritis	+	NA	Fernandes L et al 2013 (43)
Diagnostic imaging of joints in the management of rheumatoid arthritis	+	+	Colebatch AN et al, 2013 (44)
Glucocorticoid therapy in rheumatic diseases	+	NA	Duru N et al, 2013 (45)
Drug therapies in gout and hyperuricemia	+	+	Sivera F et al, 2013 (46)
Biological and non-biological therapies in rheumatoid arthritis	+	+	Smolen JS et al, 2013 (47)

*Source retrieval – from PubMed/MEDLINE. NA – not available.

It appears that better awareness of multiple facets of COI and transparent disclosure of its actual and perceived forms by all contributors of science communication may be a comprehensive solution to the existing ethical conundrums in biomedical publications. Though not always adequately perceived and reported by researchers, COI may arise at any experimental and clinical study (48). As such, reports on individual case studies and multi-center trials may equally pose ethical concerns if any conflicting relation is inappropriately disclosed. At the same time, actual COI is likely to exist in studies with large sample sizes, methodological rigor, and shiny positive outcomes (49). The prevalence of COI may also vary, depending on discipline, researchers’ geographic location, and even the corresponding authors’ gender (50). But above all, the target journal’s editorial policy with reference to best ethical standards, and strict adherence to this policy, is currently the key player in detecting and correct reporting any COI (51).

A strong foundation for ethical reporting of COI was laid down in 2010 at the 2nd World Conference on Research Integrity, which produced the Singapore Statement on Research Integrity (52). Among many points on ethical obligations of researchers and their institutions, the Singapore Statement addressed the need to declare any COI in any research document or publication, which seems a logical end-point to tireless efforts of the learned associations, aiming to implement a holistic approach to the issue of COI.

Major associations for science editors have publicized several sets of recommendations and policy papers on conflicting relations that may introduce bias in research publications (Table 3) (53-59). In this regard, perhaps the most important document is the structured COI disclosure form of the ICMJE, which was published in 2010 (54) and endorsed by most biomedical journals (60). The main advantage of the form is that it employs a list of closed, rather than open questions, correctly addressing instances of authors’ financial and other conflicts. It also sets a timeframe for existing conflicts that authors are obliged to disclose (36 months prior to publication of their papers). Notably, evidence on the implementation of the form at general medical journals such as *Deutsches Ärzteblatt*, the official organ of the German Medical Association, showed that the percentage of positive COI statements in original and review papers doubled over the initial two years of the implementation (61).

**Table 3:** Main recommendations of learned associations on conflicts of interest in biomedical publications

Associations	Documents	Year of last update	Comments	References
International Committee of Medical Journal Editors (ICMJE)	Roles and Responsibilities of Authors, Contributors, Reviewers, Editors, Publishers, and Owners: Author Responsibilities—Conflicts of Interest. ICMJE Form for Disclosure of Potential Conflicts of Interest	2010	Definition of conflict of interest, its causes and recommendations on how to disclose and report authors’, reviewers’ and editors’ potential conflicts are presented. The updated form for disclosure of conflicts of interest helps the authors to specifically address financial and other relations which may add bias in research publications.	(53,54)
World Association of Medical Editors (WAME)	Conflict of Interest in Peer-Reviewed Medical Journals	2009	The document defines conflict of interest and its different types, and provides guidance on how to disclose and manage conflicts with specific reference to the responsibilities of authors, peer reviewers and editors.	(55)
Committee on Publication Ethics (COPE)	Code of Conduct and Best Practice Guidelines for Journal Editors	2011	Journal editors are advised to implement procedures for managing their own conflicts and those of authors and reviewers.	(56)
Office of Research Integrity (ORI)	A brief overview on Conflict of Interests	2013	The guideline suggests to disclose authors’ conflicts in cover letters to journal editors and/or in footnotes of the manuscripts.	(57)
Council of Science Editors (CSE)	CSE's White Paper on Promoting Integrity in Scientific Journal Publications	2012	The Statement defines personal, financial and non-financial conflicts and guides on how to disclose them.	(58)
European Association of Science Editors (EASE)	EASE Guidelines for Authors and Translators of Scientific Articles to Be Published in English	2013	The guidelines contain publication ethics section which addresses the need to disclosure authors’ financial and personal conflicts.	(59)

In one of its major policy documents, published in 2009, the World Association of Medical Editors (WAME) recommended to report journal reviewers’ and editors’ conflicts related to the authors’ submissions, though no specific form was proposed (55). Furthermore, in its revised guidelines for editors, the Committee on Publication Ethics (COPE) advised to implement systems for managing not only authors’, but also reviewers’, and editorial staff and board members’ COIs (56). Finally, guidance from these and other associations points to the need for regularly revising journal instructions and adopting locally applicable procedures for comprehensive disclosure of COIs.

In conclusion, numerous conflicts of interest may arise at all stages of executing, reporting, and publishing biomedical research. The declaration of all relevant financial and non-financial COIs by authors of research papers is simple and remains the most important step toward the trustworthiness of science communication. Authors, reviewers, and editors should be familiar with the current research reporting guidelines, where information on funding, sources of drug supply, involvement of sponsors in research, and other ethical issues is incorporated in the checklists (62). A large number of scholarly journals have already implemented these guidelines in the process of peer review and editing. And it seems justifiable to consider the availability and explicitness of COI disclosures for journal indexing in MEDLINE and other evidence-based biomedical databases. Research institutions with their ethical committees are in a good position to improve awareness of and educate their authors on appropriate handling of all COIs. Fortunately, specialist associations are becoming more concerned with the regulation of their members’ COIs. In fact, the latest large survey of the American College of Rheumatology (771 respondents) (63) is a good example of how a clinical discipline, where COI issues remained unexplored for decades, may take the lead in curbing the ethical challenges of health professionals. Approximately 42% of the respondent rheumatologists referred to journal articles or lectures on COI and other ethical issues as the available information sources, pointing to the need for specific educational programs (63).

Strategies and effective tools for disclosing authors’ COIs are now in place in most journals, adhering to the recommendations of the ICMJE and other associations for editors. Science editors and peer reviewers, and particularly those processing reports on drug trials, systematic reviews, and practice guidelines, should carefully evaluate the authors’ reported conflicts and, when required, suggest more explicit disclosures of author-pharmaceutical industry relations in a specially designated section of the papers. The authors’ COI statements may be weighed when publishing decisions are taken although there is no data on how often journal submissions with excessive conflicts are declined. Reviewers and editors themselves may have financial and personal COIs, and it is their ethical duty to disclose any conflict to the publisher and the journal readers. There is, however, no specifically designed form for reviewers and editors, and they are usually asked to report any relevant issue throughout their work, using the online editorial management tools.

Joint efforts of authors, peer reviewers, and science editors can be a foundation for appropriately defining and disclosing potential COIs in biomedical publications.
